# A Short Review on the N,N-Dimethylacrylamide-Based Hydrogels

**DOI:** 10.3390/gels7040234

**Published:** 2021-11-26

**Authors:** Ayatzhan Akhmetzhan, Nurbala Myrzakhmetova, Nurgul Amangeldi, Zhanar Kuanyshova, Nazgul Akimbayeva, Saule Dosmaganbetova, Zhexenbek Toktarbay, Sotirios Nik. Longinos

**Affiliations:** 1 Faculty of Natural Sciecnes, L.N. Gumilyov Eurasian National University, Kazhymukan Street 5, Nur-Sultan 010008, Kazakhstan; aiatzhan@gmail.com (A.A.); dosmagambetova_ss@enu.kz (S.D.); 2 Department of Chemistry, Faculty of Natural Science, Kazakh National Woman’s Teacher Training University, Aitekebi Street 99, Almaty 700420, Kazakhstan; myrzakhmetova.nurbala@qyzpu.edu.kz (N.M.); kuanysheva.0@qyzpu.edu.kz (Z.K.); akimbayeva73@qyzpu.edu.kz (N.A.); 3 Department of Pre-University Training, Faculty of Pre-University Education, Al-Farabi Kazakh National University, Al-Farabi Av. 71, Almaty 700420, Kazakhstan; amangeldi.nurgul1@qyzpu.edu.kz; 4 Department of Chemical and Materials Engineering, School of Engineering and Digital Sciences Nazarbayev University, Kabanbaybatyr av.53, Nur-Sultan 010000, Kazakhstan; 5 Department of Petroleum Engineering, Nazarbayev University, Kabanbaybatyr av.53, Nur-Sultan 010000, Kazakhstan; s.n.longinos@gmail.com

**Keywords:** N,N-dimethylacrylamide, hydrogel, heavy metal ions sorption, self-healing, adsorption techniques

## Abstract

Scientists have been encouraged to find different methods for removing harmful heavy metal ions and dyes from bodies of water. The adsorption technique offers promising outcomes for heavy metal ion removal and is simple to run on a large scale, making it appropriate for practical applications. Many adsorbent hydrogels have been developed and reported, comprising N,N-dimethylacrylamide (DMAA)-based hydrogels, which have attracted a lot of interest due to their reusability, simplicity of synthesis, and processing. DMAA hydrogels are also a suitable choice for self-healing materials and materials with good mechanical properties. This review work discusses the recent studies of DMAA-based hydrogels such as hydrogels for dye removal and the removal of hazardous heavy metal ions from water. Furthermore, there are also references about their conduct for self-healing materials and for enhancing mechanical properties.

## 1. Introduction

DMAA is an easily polymerized, nonionic monomer. The high reactivity and low initiation temperature of DMAA make it appropriate for copolymerization. Copolymers of DMAA with N,N-dimethyl-N,N-diallylammonium chloride (DMDAAC) can be used as polymeric flocculant for water treatment [1,2,3]. The unique structure of DMAA shows amelioration in hydrogels for specific application. DMAA along with other hydrogels has a three-dimensional network structure that is capable of retaining water. In addition, DMAA hydrogel can be applied for the removal of toxic metal ions from wastewater because of characteristic properties such as chemical stability and high adsorption capacity. DMAA can also be used for flocculation due to its ability to augment the molecular weight of the copolymers.

The polymer bridging mechanism is the main flocculation mechanism. Longer polymer chains can come in contact with more colloidal particles in suspension that makes longer bridges that were built among particles, which subsequently aggregate into large flocs. The bridging effect depends on the structure of polymers. Having no intermolecular hydrogen bond, DMAA has a higher possibility of binding colloidal particles that will enhance the bridging effect. That enhanced bridging effect plays an important role in flocculation. Thus, grafted polymers of DMAA have a higher flocculation effect than polyacrylamide-grafted ones [4,5,6,7,8]. The amide group of the DMAA polymer also increases the flocculation of humic acid at very low concentration [9].

Free-radical initiated polymerization of DMAA, with a cross-linker, is frequently used for DMAA-based hydrogel preparation. Initiation is most often carried out by free-radical polymerization methods. Usually, the solution polymerization of DMAA is carried out with a water-soluble cross-linker, such as methylene bis-acrylamide (MBA). Different studies of DMAA hydrogels use cross-linkers from 0.7% to 3.2% (of total monomer weight) and initiators in the range of 0.3–1.0% in different applications. For the initiation of DMAA polymers, either ammonium or potassium persulfate are used in water media [10,11,12].

Metal ions are significant in various biochemical processes for living organisms to balance the function of biological systems. For example, zinc is both crucial for the development of the immune system and against viral infections. According to recent studies, consuming 40 mg of zinc per day could be helpful against SARS-CoV-2 infection. Trace metals (zinc, selenium, copper, magnesium) maintain immune system cells, and their deficiency can make people more vulnerable to infectious infections [13,14]. Calcium in the food can accrue the benefits of vitamin D and lowers the risk of breast cancer. On the other hand, bone diseases can be caused by a lack of calcium or deficiency of vitamin D. Although calcium can lower the risk of a variety of illnesses, it can simultaneously increase the risk of acute gastrointestinal events, kidney stones, and cardiovascular disorders including myocardial infarction and stroke [15]. Iron is the key element in the hemoglobin, playing an important role for oxygen transport [16,17]. Clinical trials have shown that copper can minimize the bacterial and viral infections, while it can also be useful in preventing the spread of infectious illnesses [14,18]. However, the presence of an excessive amount of metal ions has harmful effects on human health and the environment [19,20,21,22].

The heavy metal ions removal properties of copolymers of DMAA with 2-acrylamido-2-methylpropane sulfonic acid (AMPS) was also examined in many studies [23,24,25]. Furthermore, because its volume phase transition temperature (304–307 K) is near that of poly(N-isopropylacrylamide), N,N-dimethylacrylamide is a strong candidate for replacing poly(N-isopropylacrylamide) (307 K) [26].

## 2. N,N-Dimethylacrylamide-Based Hydrogels for Dye Removal

Many dyes and organic molecules are common cationic hazardous chemicals that must be eliminated in a wastewater treatment facility. Removing dyes from wastewater is very important due to their high toxicity for human health. If the dyes enter into the human body, they can produce bioaccumulation and augment carcinogenicity. Organic dyes not only cause damage to human health but also reduce the photosynthesis by preventing the penetration of light through water, which can negatively impact on the quality of water bodies [27,28,29,30]. Recently, scientists have developed many methods to remove dyes from water. One of the prominent ways for eliminating dyes from wastewater is hydrogel techniques. There are many hydrogels that have been used for adsorbing dyes from water such as N,N-dimethylacrylamide (DMAA).

**Figure 1:**
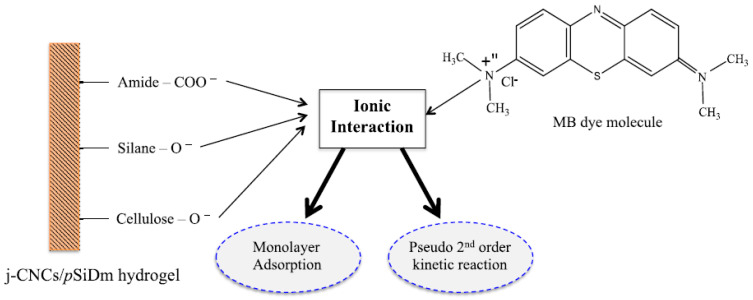
Possible adsorption mechanism of MB onto j-CNCs/pSiDm hydrogel [31].

N,N-dimethylacrylamide produces hydrogel when polymerized with cross-linkers. Moreover, poly(N,N-dimethylacrylamide) has gotten a lot of attention as it is commonly used as the hydrophilic side of copolymers due to its unique properties and high water solubility. In addition, van der Waals interactions between N,N-dimethylacrylamide and dye molecules even more increase the applicability of DMAA hydrogels. Hossain et al. proved this theory when they synthesized DMAA-based 3-methacryloxypropyltrimethoxysilane hydrogels and used them for removing methylene blue cationic dye from waste water. The maximum adsorption value of the DMAA-based hydrogel was 131.58 mg/g [31]. Since DMAA acts as a hydrogen bond acceptor, it could help to enhance the material’s adsorption properties. Apart from hydrogen bonding, the dipole–dipole interactions of amide groups of the DMAA hydrogels can also play a big part on the adsorption of methylene blue and toluidine blue dyes. Preetha and Vishalakshi synthesized Karaya gum-grafted-poly(N,N-dimethylacrylamide) hydrogel and studied the sorption of four different cationic dyes: methylene blue, crystal violet, rhodamine, and toluidine blue on the hydrogels at different pH levels. The findings of this study proved that the adsorption capacity of DMAA-based hydrogel was very high compared to many other adsorption materials [32]. The adsorption capacity of DMAA hydrogels augments with the increasing of pH of the solution due to the hydrolysis and protonation. Mechanisms of adsorption have been noticed on the works of Hossain et al. (Figure 1) [31,33]. At low value of pH, dimethyl acrylamide groups are protonated, and a positively charged NH(CH_3_)_2_^+^ surface leads to electrostatic interaction with the negatively charged dye molecules, thus improving the negatively charged dye removal [34,35].

**Figure 2:**
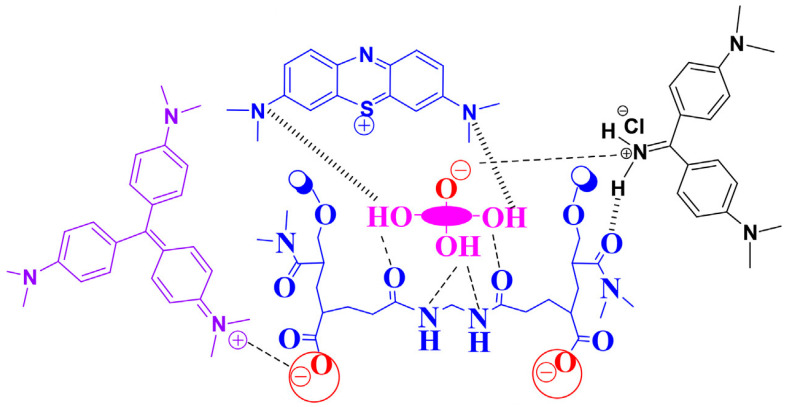
Adsorption mechanism of cationic dyes: methylene blue, crystal violet, and auramine on the DMAA-based hydrogels [36].

The synthesized DMAA hydrogels were seen to be extremely effective in removing cationic dyes due to electrostatic interaction and hydrogen bonding in the DMAA backbone. The formation of hydrogen bonding between –NH- groups in DMAA with the electronegative N in dye molecules as well as electrostatic attractions between cationic groups of dye molecules and carbonyl groups were the main driving force behind its attraction. The mechanisms of interactions are presented in Figure 2 [36,37].

Comparison of different adsorbents of N,N-dimethylacrylamide-based hydrogel for the adsorption of methylene blue and crystal violet dyes was given in Table 1.

The amounts of adsorbed dye per unit mass of hydrogel at any time t (Q_t_, mg ∗ g^−1^) and at equilibrium (Q_e_, mg ∗ g^−1^) are calculated by using the following equations:

(1) $$Q_{t\;\left({\mathrm{mg}\;*\;g}^{-1}\right)}=\frac{(C_{0}-C_{t}){\;*\;V}_{\left(L\right)}}{m_{\left(g\right)}}$$

(2) $$Q_{e\left({\mathrm{mg}\;*\;g}^{-1}\right)}=\frac{(C_{0}-C_{e}){\;*\;V}_{\left(L\right)}}{m_{\left(g\right)}}$$

where C_0_ and C_e_ are the initial and equilibrium dye concentrations (mg L^−1^), respectively, C_t_ is the dye concentration at time t, V is the volume of the solution added (L), and m is the amount of hydrogel (g).

## 3. The Removal of Hazardous Heavy Metal Ions

The removal of hazardous heavy metal ions from the environment and living organisms has recently received a lot of attention. The ease of synthesis, adaptability, abundance of raw materials, and availability of functional groups are all advantages of hydrogels. Heavy metal ions have been detected using hydrogels in extensive investigations; however, few experiments have been undertaken to remove heavy metal ions from specified systems. Furthermore, polymeric hydrogels are another potential material for removing heavy metal ions in a controlled manner in response to changes in external stimuli such as temperature, pH, electric field, and chemicals [39].

**Figure 3:**
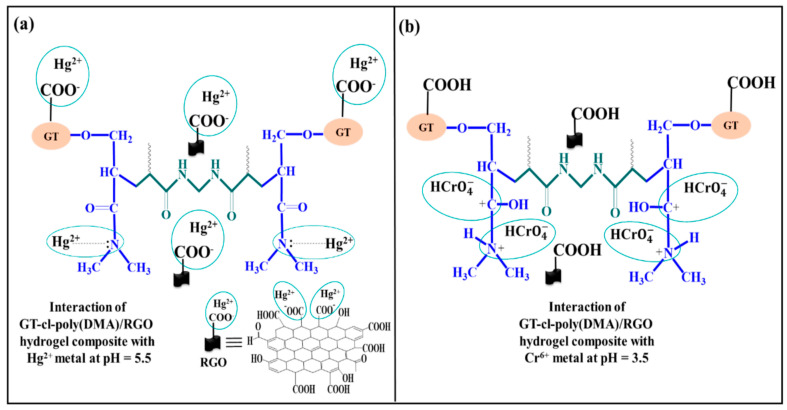
Possible interactions of DMAA hydrogel composite adsorbent with heavy metals at different pH [44].

Due to the best swelling three-dimensional crosslinked porous structure and low cost, DMAA is selected as a preferable selection for water purification. In recent studies, DMAA hydrogel nanocomposites have been used as adsorbents to remove heavy metals from wastewater. Some studies reported that DMAA hydrogels are best for removing gold ions, because their amine groups are very active for Au(III) ions [40,41]. On the other hand, at high pH, amine groups acting as a base increase the binding potentials of heavy metal cations due to the electrostatic attraction of the ions, thus improving the removing efficiency of heavy metal ions (Figure 3b) [42,43]. At low pH values, the amine group protonates and easily adsorbs the Cr^6+^ ions in the form of HCrO_4_^−^ (Figure 3a). The mechanism of the adsorption is given in Figure 3 [44].

Since N,N-dimethyl groups in DMAA are less likely to produce quaternary ammonium cation at low pH, the impact of pH is negligible for DMAA hydrogels [45]. DMAA hydrogels’ low pH sensitivity allows them to absorb metal ions at various pH values. In addition, unbonded electrons of nitrogen and oxygen in DMAA hydrogel can help form complexation with metal ions with empty orbitals. Thus, there is an increase in the sorption capacity [46,47]. The comparison of different adsorbents of N,N-dimethylacrylamide-based hydrogel for the adsorption of different metal ions are presented in Table 2. The amounts of adsorbed metal ions per unit mass of hydrogel at any time t (Q_t_, mg ∗ g^−1^) and at equilibrium (Q_e_, mg ∗ g^−1^) are calculated by using Equations (1) and (2).

## 4. N,N-Dimethylacrylamide Hydrogels for Self-Healing Materials

Self-healing hydrogels are a form of polymer hydrogel that has the ability to heal itself. Self-healing is the formation of new bonds when old bonds are broken within a material. Electrostatic attraction forces between molecules can create the formation of new bonds by reconstructive covalent side chain or non-covalent hydrogen bonding. These properties of self-healing hydrogel have attracted attention in many fields. Hydrogels with self-healing, injectable, and stimuli-responsive properties can have many advantages. For example, self-healing hydrogels implanted in the body can form their original state after damaging by external forces. This increases the body’s safety and reduces the economic cost [51]. On the other hand, self-healing hydrogels easily release and carry biological active compounds [52].

**Figure 4:**
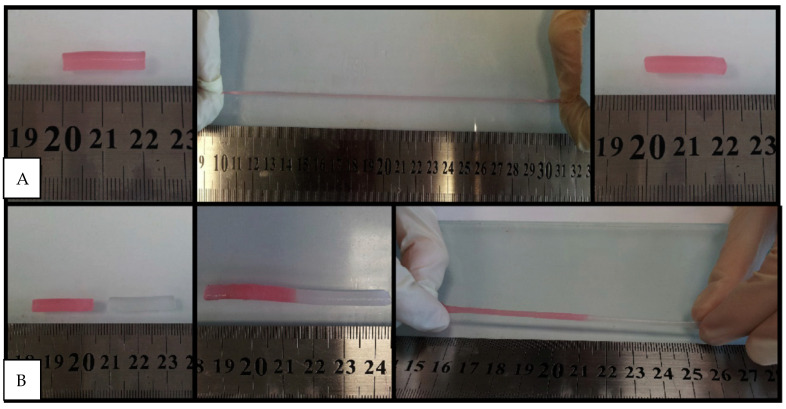
(**A**): Photographs of a gel sample before and after stretching to an elongation ratio of 10. After a waiting time of 10 min, it recovers its original length. (**B**): Photographs of two gel samples. One of the samples was colored with a dye for clarity. After cutting into two pieces and pressing the fractured surfaces together for 10 min, they merge into a single piece [56].

The hydrogen bond and electrostatic attraction forces between N,N-dimethylacrylamide molecules can hold and easily release bioactive compounds (drugs) depending on the medium of the body. Du et al. prepared N,N-dimethylacrylamide-stat-3-acrylamidophenylboronicacid statistical copolymers (PDMAA-stat-PAPBA) and poly(glycerolmonomethacrylate) (PGMA) chains grafted cellulose nanocrystals (CNC-g-PGMA) and studied the mechanical and self-healing properties of the new hydrogel. According to the study, the self-healing properties were enhanced by adding N,N-dimethylacrylamide. Self-healing N,N-dimethylacrylamide hydrogels are not only drug carrier but also can be used in biosensors. Hou et al. developed N,N-dimethylacrylamide self-healing hydrogels with thermo-responsive properties that can be used in medicine as a body temperature regulator [53]. The unique structure of N,N-dimethylacrylamide hydrogels is mainly due to having both hydrophobic interactions and hydrogen bonding in its hydrogels [54]. The hydrogen bonding and hydrophobic interactions between the methyl groups of the N,N-dimethylacrylamide network are responsible for the reentrant transition conduct of the hydrogels. The hydrogels hold up to about 4200% strain, and the damage created in the gels could be healed at 50 °C within 10 h (Figure 4A,B) [55,56].

## 5. N,N-Dimethylacrylamide Hydrogels for Enhancing Mechanical Properties of the Materials

**Figure 5:**
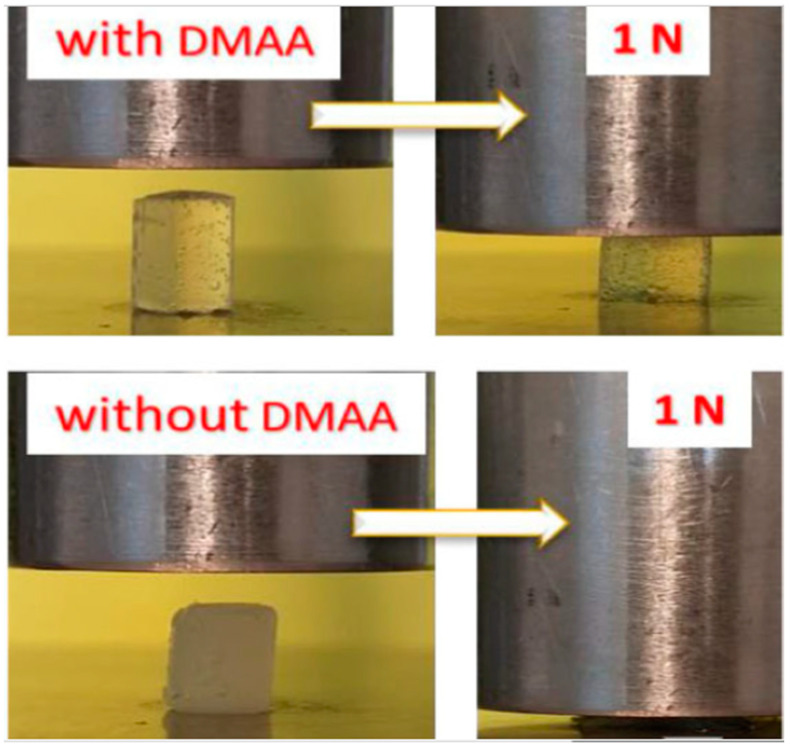
Compression of two cylindrical hydrogel specimens prepared without and with DMAA under a nominal stress of 63 kPa [60].

The hydrophobic interactions, hydrogen bonding, and ion–dipole interactions in the single structure of N,N-dimethylacrylamide can augment the mechanical strength of hydrogel. Weng et al. modified natural polysaccharides with DMAA and obtained highly porous hydrogels with high mechanical strength. Even though the obtained hydrogel contained more than 90% water, it still withstood high compressive strength [57,58,59]. Furthermore, in Figure 5, hydrogels with and without the addition of DMAA were demonstrated. Despite the fact that they were both of the same toughness, the one containing DMAA was deformed by 26% without producing noticeable damage, whilst the other was broken entirely. In DMAA-containing hydrogels, the energy dissipation process inhibits force localization and therefore prevents the hydrogel matrix from macroscopic damage [60,61,62].

Pendant vinyl groups in DMAA monomer can be incorporated into other monomers as a spacer and can increase the flexibility of hydrogels [63,64,65,66]. C.B. Oral et al. included DMAA monomer into silk fibroin hydrogel and changed the brittle hydrogel into a stretchable hydrogel with an elongation ratio up to 370% [60].

## 6. Synthesis of DMAA-Based Hydrogels

Developments in the design of hybrid DMAA-based hydrogels materials that may be employed for efficient dye and heavy metal removal from waste water will be a priority for us. Tokuyama et al. prepared highly crosslinked DMAA gel, which was composed by DMAA/MBAA = 750/30 mol/m^3^, N,N-methylenebisacrylamide (MBA) as cross-linker (30 mol/m^3^), N,N,N,N-tetramethylethylenediamine as catalyzer (5 mol/m^3^), and ammonium peroxodisulfate (APS) (0.5 mol/m^3^) as initiator. The reaction was carried out at 5 °C under nitrogen atmosphere for 24 h. The resulted hydrogel was used for AU(III) ions detection [40]. A XG-cl-DMAA/SiO_2_ hydrogel nanocomposite was synthesized by using MBA as the cross-linker and APS as the initiator in a domestic microwave. For XG-cl-DMAA/SiO_2_ hydrogel synthesis, 0.1 g of xanthan gum (XG) was dissolved in 100 mL of DI water, and DMAA along with MBA were added. Then, APS was added as an initiator for graft copolymerization. Afterwards, 0.1 g of SiO_2_ in 5 mL of DI water was added. The hydrogel was precipitated with acetone. The adsorption kinetics and isotherms of Cd^2+^ were calculated [48]. The same procedure was used for the synthesis of gum tragacanth-cl-N,N-dimethylacrylamide (GT-cl-poly(DMAA)) hydrogel [44]. In similar way, starch-grafted poly(N,N-dimethyl acrylamide) hydrogel was synthesized. In a starch-grafted poly(N,N-dimethyl acrylamide) hydrogel experiment, 1 g from a 40 mL water solution of starch was added both into different amounts (2.5–10 g) of DMAA and MBA (0.002–0.006 g), while 0.3 g of APS was used as an initiator [35]. Akhmetzhan et al. synthesized DMAA hydrogel with Na-AMPS at different molar ratios of monomers. APS initiator was used at different concentrations from 0.05 to 0.1 wt % of total monomer mass along with 1.5–8.5 wt % cross-linker (MBA). Kinetics of copolymerization were calculated at different monomer and initiator concentrations. In addition, the removal of heavy metal ions was evaluated depending on the composition of hydrogel [25,67,68,69]. H. Tokuyama et al. synthesized DMAA hydrogel with zirconia nanoparticles by photoinitiated polymerization using MBAA as a crosslinking agent and 2,2’-azobis(2-methylpropionamidine) dihydrochloride as an initiator. The polymerization was initiated by UV irradiation in a tube at 298 K for 24 h under nitrogen atmosphere. The prepared hydrogel was used for an arsenic sensor [41,42].

Poly(N,N-dimethylacrylamide-*co*-acrylamide)-grafted hydroxyethyl cellulose (HEC-g-(PAM-*co*-PDMAA)) hydrogel was prepared by Jana et al. The polymerization was initiated by K_2_S_2_O_8_ (KPS) and crosslinked by MBA. A typical synthetic procedure was as follows: 2.0 g of HEC was dissolved in 100 mL of distilled water in a 250 mL conical flask. Then, 6 mL of DMAA, 6 g of AM, and 0.1 g MBA were added one after another in the HEC at 70 °C. Then, KPS initiator was added in the reaction mixture. The dried hydrogel was used as Congo red dye remover [34,36]. Poly(N,N-dimethylacrylamide-*co*-sodium acrylate) hydrogel, P(DMAA-*co*-ANa), was synthesized by radical polymerization. In this process, 0.25 g of methylene bisacrylamide in 40 mL water was added into the mixture of N,N-dimethylacrylamide (0.025 mol) and acrylic acid (0.025 mol). After adjusting the pH between 5 and 6, 0.1 g of ammonium persulfate and 0.1 g of N,N,N_,N_-tetramethylene diamine were added. The reaction was left to proceed for 24 h, and the gel was neutralized by NaOH 1 M solution (pH 13). Then, it was soaked in pure water for 1 week. Water was renewed daily. The obtained material was used for removing cationic dye and metal ions [38].

The synthesized hydrogels were characterized by Fourier transform infrared spectroscopy (FTIR). The morphology of hydrogels was determined by scanning electron microscope (SEM), while chemical composition was confirmed by X-ray photoelectron spectroscopy (XPS). Moreover, the thermal behavior of hydrogels was examined by differential scanning calorimetry (DSC) and thermogravimetry (TG).

## 7. Conclusions

There are many methods of removing heavy metals and organic dyes from water. Among them, adsorption by DMAA hydrogels is a simple, easy, and cheap method. In this review, many methods for the preparation of DMAA-based hydrogel were presented. The selected studies focused on improving DMAA hydrogel for the effective removal of heavy metal ions and organic dyes from polluted water. Comparisons were made on the adsorption of different metal ions and dyes by DMAA hydrogels. Based on the possible interactions between DMAA adsorbents and heavy metal ions, new ways of modification of DMAA hydrogels for removing more heavy metal ions and dye molecules were presented by different researchers. According to the research in this review, DMAA copolymerized with hydroxyethyl methacrylate has the highest metal ions absorption values for Pb(II) ions, with maximum absorption capacities of 70.52 mg/g, while the maximum absorption value of DMAA grafted with hydroxyethyl starch for Pb(II) ions was 51.75 mg. The explanation for this outcome was due to carboxylic group ions in hydroxyethyl methacrylate that might attract more metal ions than intermolecular interactions in the starch. Hence, some research groups increased electrostatic interactions by the inclusion of graphene oxide and tragacanth gum into DMAA matrix. This fact led to the creation of the highest metal ions sorption such as 416.66 mg/g for Cr(IV) ions, while DMAA-N-vinylcaprolactam-g-chitosan had maximum absorption capacities of 142.86 mg/g.

The synthesized DMAA hydrogels were seen to be extremely effective in removing cationic dyes due to electrostatic interaction and hydrogen bonding in the DMAA backbone. The formation of hydrogen bonding between –NH- groups in DMAA with the electronegative N in dye molecules as well as electrostatic attractions between cationic groups of dye molecules and carbonyl groups were the main driving force behind its attraction. Furthermore, poly(N,N-dimethylacrylamide-co-sodium acrylate) has the highest adsorption capacity for methylene blue and crystal violet dyes, with adsorption capacities of 800 mg/g and 320 mg/g, respectively. On the other hand, karaya gum-grafted-DMAA gel showed the lowest value, for methylene blue 11.93 mg/g and for crystal violet 41.84 mg/g. These results indicated that the inclusion of molecules into the DMAA matrix with a high swelling ratio can increase the sorption capacity.

According to the analyses in this review article, DMAA-based hydrogel adsorbents often remove specific ions. It is impossible to remove all the metal ions with DMAA-based hydrogel adsorbents. Thus, more research should be focused on developing effective adsorbents for the full removal of several heavy metal ions and dyes.

## Figures and Tables

**Table 1:** Comparison of different adsorbents of N,N-dimethylacrylamide-based hydrogel for the adsorption of methylene blue and crystal violet dyes.

№	Name of the Hydrogel	The Adsorption Capacity, Qe (mg/g)		References
		Methylene Blue	Crystal Violet	
1	poly(N,N-dimethylacrylamide-co-sodium acrylate)	800	320	[38]
2	katira gum-cl-poly(acrylic acid-co-N,N-dimethylacrylamide)@bentonite	165.28	158.73	[34]
3	poly(N,N-dimethylacrylamideco- 2-hydroxyethyl methacrylate)	80.27	-	[37]
4	poly(N,N-dimethylacrylamide-co-3-methacryloxypropyltrimethoxysilane)	131.58	-	[31]
5	karaya gum-grafted-poly( N,N-dimethylacrylamide) gel	11.93	41.84	[32]

**Table 2:** Comparison of different adsorbents of N,N-dimethylacrylamide-based hydrogel for the adsorption of different metal ions.

№	Name of the Hydrogel	Metal Ions	The Adsorption Capacity, Qe (mg/g)	References
1 2 3	N,N-dimethylacrylamide-co-sodium acrylate	Cr(III)	450	[38]
		Co(II)	300	
		Ni(II)	298	
4 5	graphene oxide incorporated gum tragacanth-cl-N,N-dimethylacrylamide (GT-cl-poly(DMAA)/RGO)	Hg(II)	636.94	[44]
		Cr(VI)	416.66	
6	N,N-dimethylacrylamide-co-2-hydroxyethyl methacrylate	Pb(II)	70.52	[46]
7	xanthan gum-cl-Dimethyl acrylamide hydrogel containing silica	Cd(II)	150.7	[48]
8			50.35	
9	N,N-dimethylacrylamide- N-vinylcaprolactam-g- Chitosan	Cr(VI)	142.86	[49]
10	N,N-dimethyl acrylamide-g- Hydroxyethyl starch	Hg(II)	300	[50]
		Cu(II)	80.6	
		Zn(II)	64	
		Pb(II)	51.75	

## Data Availability

Data is contained within the article.
